# Antisense DNA parameters derived from next-nearest-neighbor analysis of experimental data

**DOI:** 10.1186/1471-2105-11-252

**Published:** 2010-05-14

**Authors:** Donald M Gray, Carla W Gray, Byong-Hoon Yoo, Tzu-Fang Lou

**Affiliations:** 1 Department of Molecular and Cell Biology, The University of Texas at Dallas, 800 W. Campbell Road, Richardson, Texas, 75080, USA; 2 Departments of Pediatrics and Biochemistry and Molecular Biology, Atlantic Research Center, Room C-302, Dalhousie University, 5849 University Avenue, Halifax, Nova Scotia, B3H 4H7, Canada

## Abstract

**Background:**

The enumeration of tetrameric and other sequence motifs that are positively or negatively correlated with *in vivo *antisense DNA effects has been a useful addition to the arsenal of information needed to predict effective targets for antisense DNA control of gene expression. Such retrospective information derived from *in vivo *cellular experiments characterizes aspects of the sequence dependence of antisense inhibition that are not predicted by nearest-neighbor (NN) thermodynamic parameters derived from *in vitro *experiments. However, quantitation of the antisense contributions of motifs is problematic, since individual motifs are not isolated from the effects of neighboring nucleotides, and motifs may be overlapping. These problems are circumvented by a next-nearest-neighbor (NNN) analysis of antisense DNA effects in which the overlapping nature of nearest-neighbors is taken into account.

**Results:**

Next-nearest-neighbor triplet combinations of nucleotides are the simplest that include overlapping sequence effects and therefore can encompass interactions beyond those of nearest neighbors. We used singular value decomposition (SVD) to fit experimental data from our laboratory in which phosphorothioate-modified antisense DNAs (S-DNAs) 20 nucleotides long were used to inhibit cellular protein expression in 112 experiments involving four gene targets and two cell lines. Data were fitted using a NNN model, neglecting end effects, to derive NNN inhibition parameters that could be combined to give parameters for a set of 49 sequences that represents the inhibitory effects of all possible overlapping triplet interactions in the cellular targets of these antisense S-DNAs. We also show that parameters to describe subsets of the data, such as the mRNAs being targeted and the cell lines used, can be included in such a derivation. While NNN triplet parameters provided an adequate model to fit our data, NN doublet parameters did not.

**Conclusions:**

The methodology presented illustrates how NNN antisense inhibitory information can be derived from *in vivo *cellular experiments. Subsequent calculations of the antisense inhibitory parameters for any mRNA target sequence automatically take into account the effects of all possible overlapping combinations of nearest-neighbors in the sequence. This procedure is more robust than the tallying of tetrameric motifs that have positive or negative antisense effects. The specific parameters derived in this work are limited in their applicability by the relatively small database of experiments that was used in their derivation.

## Background

Antisense oligodeoxynucleotides are typically targeted to bind mRNA sequences, leading to inhibition of gene expression by activation of RNase H to cleave the mRNA, obstruction of translation, alteration of splicing, or other mechanisms. The experimental determination of an effective antisense DNA to inhibit the expression of a particular gene product is expensive and time-consuming, and efforts have long been made to develop a procedure for the rational design of antisense DNA sequences based on properties such as the DNA:RNA hybrid stability, the region of the mRNA being targeted, and the secondary structures of the mRNA and DNA (reviewed by Chan et al. [1]). Programs using *in vitro *thermodynamic information for intrastrand and interstrand DNA and RNA interactions can be used to help discriminate weak from potent antisense DNA sequences [2,3]. While extremely important for understanding stabilities of base pairs *in vitro*, the underlying thermodynamic information in such programs (e.g. the *RNAstructure *program at http://rna.urmc.rochester.edu/RNAstructure.html[4,5]) is limited in its use for predictions of hybridization stability under intracellular conditions. Thermodynamic data have been typically obtained for standard Watson-Crick base pairs in unmodified nucleic acids under non-physiological solution conditions, such as in the presence of 1 M NaCl and in the absence of proteins and enzymes that bind to nucleic acids. Most *in vitro *thermodynamic data are adequately modeled by the assumption that stabilities arise from interactions between adjacent base pairs and therefore are nearest-neighbor in origin [6-8]. However, Owczarzy et al. [8] have shown that there is a significant enthalpic contribution to the stability of double-stranded DNAs from NNN base pair triplets when the Na^+ ^ion concentration falls below 55 mM, and the effect is sequence-dependent. The range of magnitudes of these NNN triplet contributions is up to about 1/3 of those of the NN doublet contributions.

A new concept in the design of effective antisense DNAs was introduced by Tu et al. [9], who reported that DNAs containing a TCCC tetranucleotide motif, complementary to GGGA in mRNA transcripts, were above average in their ability to downregulate tumor necrosis factor-α synthesis. That work pointed to the possible existence of important sequence-dependent interactions that extend beyond the nearest-neighbors and that influence antisense efficacy. Moreover, implicit in this work was the concept that the analysis of experimental data from antisense treatments of cells could yield sequence-dependent information that might be more inclusive than nearest-neighbor stabilities derived from *in vitro *measurements.

Further studies have identified many other nucleotide motifs that are positively as well as negatively correlated with antisense nucleotide activity [10-12]. From an analysis of 3913 S-DNA sequences, McQuesten and Peek [11] reported 155 motifs of 2 to 5 nucleotides associated positively and 202 motifs associated negatively with antisense effectiveness. Sipes and Freier [12] used a proprietary database of over 12,000 antisense DNAs of all types to derive a more limited set of tetrameric motifs, presented as a few "aggregate motifs" with flexible base designations. These aggregate motifs were a summary of 24 tetramers that were positively correlated with an inhibitory effect and 20 tetramers that were negatively correlated with antisense inhibition. The identification of motifs in an antisense DNA sequence does not by itself allow a confident prediction of antisense effectiveness, but the numbers of positive and negative motifs can be combined in a serial fashion with other attributes in an "if-then" decision tree to give significantly enhanced predictions of antisense effectiveness of various DNA sequences [12].

The reasons for the existence of motifs associated positively or negatively with antisense effectiveness are unknown. Interactions that are NNN in extent could play a role, but other effects might be even more important within the cell. Most of the work to identify motifs has been done with antisense oligomers containing phosphorothioate linkages to inhibit DNase degradation and still allow RNase H activity, and the possibility of RNase-dependent sequence specificity has been suggested [9]. Proteins that bind and sequester single-stranded DNA sequences and inhibit productive mRNA binding could be involved. Other possible reasons for the longer-range sequence effects expressed as motifs have been discussed by Tu et al. [9].

While valuable as an adjunct to other attributes of antisense efficacy, motifs identified as being either simply positive or negative in their effects are limited in their predictive utility. Motifs of any length greater than NN doublets have overlapping interactions within a sequence, so that their combined effects are difficult to quantitate. For example, positive tetramer motifs of (G/A)(G/A)CA have a CA overlap with other positive motifs of CAG(G/C) (with the motifs written as mRNA sites), and positive motifs of (G/C)AGC have a GC overlap with other positive and negative motifs (respectively GCA(G/U) and GC(G/C)C) [12]. In general, it is to be expected that attributes assigned to motifs will be influenced by adjacent sequences.

The present work illustrates how a NNN model can be used to derive parameters that may more completely encompass the sequence dependence of results from *in vivo *antisense DNA experiments. The parameters derived from such a model can be used to obtain an unambiguous value for the inhibitory potential of any relevant mRNA target sequence (or complementary antisense DNA sequence), containing any combination of overlapping next-nearest-neighbors.

## Results

### NNN parameters for mRNAs targeted by antisense S-DNAs

A data set of 112 antisense experiments, using 20-mer antisense S-DNAs, two cell lines and four gene targets (see Methods), was analyzed by singular value decomposition (SVD) [13] to determine the values of NNN triplet (trinucleotide sequence) parameters, P, and parameters associated with differences due to the cell line and gene targeted, that best fit the experimental results. As explained under Methods, a simplifying assumption made in the analysis was that the mRNA targets of the S-DNA sequences were closed circular sequences 20 nucleotides long. A statistical test showed that the NNN model including the cell and gene parameters was acceptable, in that the probability Q that the observed χ^2 ^from a fit to the data would be larger by chance was 0.33 (a larger Q indicates a better fit; values of Q > 0.1 are adequate) [13]. An SVD analysis in which the cell parameter was omitted gave a worse fit, as expected, with a Q value of 0.0013. Table 1 lists a (non-unique) set of derived parameters, P (numerical values of the percent change in the amount of cellular protein) associated with all 64 NNNs in the mRNA target. Although these derived values are generally not meaningful in terms of the individual NNN, their sum does give a unique value for the predicted change in net expressed protein when they are combined into a complete closed circular mRNA target sequence (13,14). A positive sum indicates an inhibition of the amount of accumulated protein, while NNNs with negative values act to reduce the inhibitory effect when they are present in a target mRNA sequence.

**Table 1:** 64 mRNA triplets and associated inhibition parameters, P.

NNN triplet or other parameter (5' to 3' in mRNA)	Values of antisense inhibition parameter P (%) from fit to 112 sequences ± standard error from SVD analysis	Number of NNN triplet or other parameter in data set
AAA	2.98 ± 2.08	30

AAC	5.96 ± 1.45	32

AAG	0.48 ± 1.82	36

AAU	-3.52 ± 1.76	30

ACA	-3.78 ± 1.67	48

ACC	4.63 ± 2.79	25

ACG	1.82 ± 1.69	28

ACU	3.81 ± 1.66	33

AGA	3.23 ± 1.99	40

AGC	3.86 ± 1.46	39

AGG	4.83 ± 1.82	52

AGU	-4.72 ± 2.20	17

AUA	0.29 ± 2.42	15

AUC	3.27 ± 2.07	19

AUG	0.45 ± 1.63	67

AUU	1.05 ± 1.97	21

CAA	1.83 ± 1.65	37

CAC	-1.51 ± 1.31	46

CAG	-1.47 ± 1.49	38

CAU	5.77 ± 1.62	29

CCA	-0.99 ± 2.14	26

CCC	-3.73 ± 2.06	30

CCG	3.09 ± 1.28	58

CCU	5.48 ± 1.76	35

CGA	5.43 ± 1.64	40

CGC	7.38 ± 1.32	33

CGG	5.65 ± 1.49	51

CGU	-5.02 ± 1.96	19

CUA	13.27 ± 2.79	14

CUC	1.37 ± 1.75	31

CUG	0.87 ± 1.75	44

CUU	-4.62 ± 1.64	28

GAA	3.57 ± 2.00	53

GAC	1.08 ± 1.67	37

GAG	-2.19 ± 1.07	63

GAU	4.87 ± 1.71	47

GCA	6.89 ± 1.64	46

GCC	-1.26 ± 1.57	58

GCG	3.96 ± 1.59	41

GCU	6.20 ± 1.91	31

GGA	2.96 ± 1.10	82

GGC	-2.70 ± 1.10	56

GGG	2.06 ± 1.28	76

GGU	8.28 ± 1.99	30

GUA	-8.59 ± 2.99	15

GUC	1.85 ± 1.84	24

GUG	-3.40 ± 1.46	42

GUU	10.43 ± 2.95	18

UAA	-2.48 ± 3.44	8

UAC	0.94 ± 1.91	19

UAG	10.37 ± 2.55	11

UAU	-2.06 ± 2.69	16

UCA	2.50 ± 1.75	30

UCC	4.20 ± 1.98	36

UCG	4.57 ± 2.47	16

UCU	-4.60 ± 2.60	18

UGA	-4.28 ± 1.93	38

UGC	7.26 ± 1.14	48

UGG	-1.93 ± 1.33	65

UGU	1.75 ± 2.00	33

UUA	1.80 ± 3.21	10

UUC	0.19 ± 2.06	26

UUG	4.88 ± 2.28	31

UUU	0.81 ± 2.59	25

A549 cell	10.59 ± 1.78	62

CRAF1	3.65 ± 2.33	52

BCL2	-3.10 ± 1.85	29

AKT2	8.97 ± 4.43	7

PKC-α	-3.75 ± 2.76	24

Values of parameters, P, for 64 NNN mRNA triplets obtained from an SVD solution of inhibitory data for 112 antisense S-DNA sequences targeted to mRNAs encoded by four genes, in two cell lines. Except for the NNN parameters for AAA, UUU, CCC, and GGG, and the parameter for the cell line, the parameters are meaningful only in combinations. The parameters are the percent changes in net protein accumulation assigned to the NNN such that they may be summed to give inhibitory values for closed-circular mRNA antisense targets. (Positive values denote a decrease in accumulated protein.)

The same data were also subjected to an analogous SVD analysis using the simpler NN model of 16 possible dinucleotide sequences, plus other parameters for the cell line and targeted genes (results not shown). The Q value from using the NN model to fit the experimental data was imperceptibly small, showing that the simpler NN model was inadequate to account for the data.

**Figure 1:**
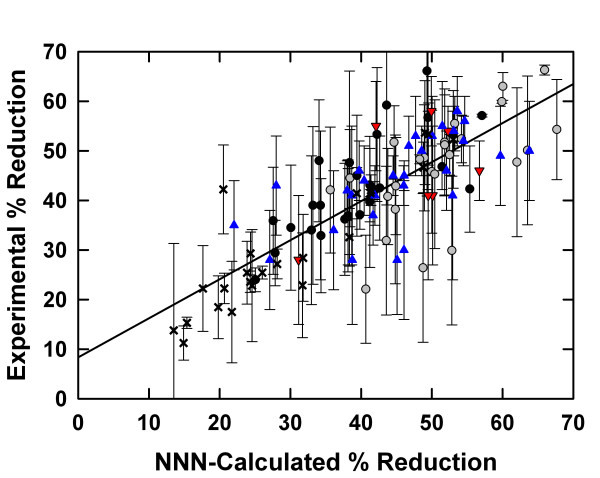
**Experimental *versus *NNN-calculated percent reduction in accumulated protein**. Experimental values of percent reduction in net protein accumulation from 112 antisense S-DNA experiments *versus *values calculated by summing NNN parameters from Table 1. The experimental values and errors are given in additional file 1. The correlation coefficient r = 0.792. Symbols are as follows: × (PKC-α), blue triangle (BCL2), red triangle (AKT2), grey circle (CRAF2 in A549 cells), black circle (CRAF2 in T24 cells).

Values of the percent reductions in accumulated protein (i.e. the percent reductions in protein accumulated in the cells in 20-24 h) for the 112 experiments were recalculated from the parameters in Table 1 and, as expected, were highly correlated with the measured experimental values (correlation coefficient r = 0.792), as seen in Figure 1.

### Parameters for independent sequence combinations

As mentioned above, the NNN triplet parameters are not independent and generally are not meaningful by themselves, with the exception of parameters for the four homotrinucleotide sequences, AAA, UUU, CCC, and GGG. That is, an actual mRNA sequence has a restricted combination of the NNNs, and, correspondingly, the parameters in Table 1 are physically meaningful only when they are combined within an actual sequence [14]. There are 15 linear equations that constrain the numbers of the NNN triplets in a closed, circular single-stranded sequence (analogous to the three equations that constrain the presence of the 16 possible NN [14]). Thus, while the parameters of Table 1 can be combined to estimate the effectiveness of an antisense S-DNA against any given target mRNA sequence (limited of course by the NNN model and the genes, cell type, and other aspects of the database), there are only 49 combinations of the 64 NNN that make up an irreducible, linearly independent set of sequences. In seeking mRNA sequences that have an array of NNN triplets that correlate with being effective antisense S-DNA targets, one has to consider combinations of the NNN parameters, not the individual parameters themselves. Although there are innumerable sets of such irreducible combinations that may be chosen, any set provides a succinct description of the sequence-dependent inhibitory properties of mRNA targets (and the complementary S-DNAs that bind to them) used in the database.

An example set of 49 independent combinations of 12-mer mRNA sequences is given in Table 2, along with their calculated parameters in descending rank. This set of sequences was chosen to be as simple as possible and includes the four homonucleotides, six repeating dinucleotides, and 20 repeating trinucleotides, with the remaining sequences being 19 repeating tetranucleotides. This set was shown to be linearly independent by singular value decomposition. (That is, a 64 row × 49 column matrix, with the numbers of NNN within the 49 sequences constituting the columns, had no zero singular values [results not shown].) The P values in Table 2 are the summed NNN values from Table 1. For example, the P value for (ACU)_4 _is P(ACU)_4 _= 4 × P(ACU) + 4 × P(CUA) + 4 × P(UAC) = 4 × (3.81 + 13.27 + 0.94) = 72.1% reduction in net protein accumulation. The standard errors in Table 2 were derived using the complete variance-covariance matrix from the SVD analysis. On average, errors for these combinations of NNN P values were 90% correct when simply calculated as the square root of the sum of squares of the errors on the P values for the component NNN (from Table 1), neglecting the covariance values. The errors on the P values for the independent combinations were significant, but they were less for the ten combinations that were ranked to have the highest inhibitory parameters (± 15% error on average) than for the ten combinations with the lowest inhibitory parameters (± 18% error on average).

**Table 2:** Inhibition parameters for linearly independent combinations of next-nearest-neighbor triplets.

Independent sequence (12-mer) (5' to 3' in mRNA)	Antisense inhibition parameter P (%) per 12-mer
(ACU)_4_	72.1 ± 14.7

(UUG)_4_	68.2 ± 21.2

(CG)_6_	68.0 ± 14.7

(AACU)_3_	61.7 ± 17.1

(GGUC)_3_	61.0 ± 11.3

(UGC)_4_	57.3 ± 9.0

(UUAG)_3_	53.6 ± 20.7

(AACG)_3_	50.3 ± 10.6

(AUC)_4_	46.2 ± 14.0

(GGUA)_3_	44.7 ± 15.6

(UCC)_4_	44.2 ± 14.0

(UUGC)_3_	41.2 ± 10.0

(AAGC)_3_	39.2 ± 7.5

(AGC)_4_	37.1 ± 10.7

(CCUG)_3_	37.0 ± 10.2

(CCG)_4_	36.8 ± 9.1

(A)_12_	35.7 ± 24.9

(GGAC)_3_	34.5 ± 8.2

(ACG)_4_	33.3 ± 11.7

(UUCG)_3_	30.5 ± 19.6

(AAG)_4_	29.1 ± 16.1

(UUCA)_3_	28.5 ± 9.5

(CGG)_4_	27.6 ± 9.2

(G)_12_	24.8 ± 15.4

(GGCA)_3_	22.7 ± 9.7

(AGG)_4_	22.4 ± 8.6

(GGAU)_3_	19.0 ± 7.5

(AAC)_4_	16.0 ± 11.2

(AAUC)_3_	12.2 ± 11.8

(UGG)_4_	11.8 ± 9.7

(U)_12_	9.7 ± 31.1

(ACC)_4_	8.5 ± 18.1

(GGCU)_3_	7.3 ± 9.1

(AG)_6_	6.2 ± 12.6

(UUAC)_3_	5.8 ± 16.7

(UCG)_4_	5.6 ± 15.6

(AUG)_4_	4.2 ± 12.8

(AUU)_4_	3.2 ± 16.0

(CCAG)_3_	0.4 ± 11.5

(UG)_6_	-9.9 ± 15.4

(AU)_6_	-10.6 ± 26.0

(AAUG)_3_	-11.3 ± 10.1

(AGU)_4_	-11.8 ± 15.9

(UC)_6_	-19.4 ± 19.8

(AAU)_4_	-22.8 ± 17.7

(AC)_6_	-31.8 ± 12.4

(UUC)_4_	-36.1 ± 14.9

(C)_12_	-44.8 ± 24.7

(AAGU)_3_	-46.0 ± 20.5

Values of inhibition parameters, P (percent change, with positive values indicating a decrease in accumulated protein) for an example set of 49 linearly independent 12-mer mRNA sequence combinations of NNN triplets. Values are ranked in decreasing order. Percent standard errors were derived from the complete SVD variance-covariance matrix (i.e. including the covariances).

One aspect of the ranking of the P values for the independent sequences in Table 2 is in general agreement with known stabilities of S-DNA:RNA and DNA:RNA hybrids *in vitro*. The independent mRNA sequences that contain only purines are generally ranked as more favorable targets than matching sequences that contain only pyrimidines, as shown in Table 3. (An exception in this table is that (UCC)_4 _has a larger P value than (AGG)_4_.) This asymmetric pattern of hybrid stabilities undoubtedly has its origin in the stabilities of NN interactions and has been documented in studies of S-DNA:RNA and DNA:RNA oligomer hybrids, with hybrids being thermally and thermodynamically more stable when the RNA strand has a dominant purine content, for sequences of the same A + U(T) content [6,15,16].

**Table 3:** Inhibition parameters for independent sequence combinations of NNN that contain only purines or pyrimidines.

Parameters P (%) for five purine-containing independent sequences		Parameters P (%) for five pyrimidine-containing independent sequences	
(A)_12_	35.7 ± 24.9	(UCC)_4_	44.2 ± 14.0

(AAG)_4_	29.1 ± 16.1	(U)_12_	9.7 ± 31.1

(G)_12_	24.8 ± 15.4	(UC)_6_	-19.4 ± 19.8

(AGG)_4_	22.4 ± 8.6	(UUC)_4_	-36.1 ± 14.9

(AG)_6_	6.2 ± 12.6	(C)_12_	-44.8 ± 24.7

Inhibition parameters, P, from Table 2 for the five independent sequence combinations that contain only purines and the five that contain only pyrimidines. The former are generally more favorable target sequence combinations than those containing only pyrimidines.

### Parameters for cell type and targeted gene

Cell- and gene-specific parameters were included in the SVD analysis of the reductions in accumulated protein according to the NNN model. A549 cells were more sensitive targets than were T24 cells by 10.6 ± 1.8% in the extent of protein inhibition (Table 1). With respect to the mRNAs of the four targeted genes, four parameters were derived (Table 1), but only three of these four parameters were independent. (This is because only one of the genes could be specified for each targeted mRNA sequence, which constrained the sum of occurrences of the four targeted genes to be 1.0 in any given row of the matrix of values, **N**_h, k_, that was analyzed by SVD. See Methods.) One way of expressing the inhibition parameters for specific genes is in terms of their differences from the average (Table 4). Then, any three of the difference parameters make an independent set, with the fourth being a dependent parameter that is the negative sum of the three chosen to be independent. The inhibition parameters of Table 4 show that protein accumulation from either the CRAF1 or AKT2 mRNA targets was reduced more than the average, while the accumulation of protein from either the BCL2 or PKC-α mRNA targets was reduced less than the average.

**Table 4:** Inhibition parameters for independent combinations of targeted genes.

Parameters for each of four gene combinations	Antisense inhibition parameter P (%)
CRAF1 minus average	2.20 ± 2.36

BCL2 minus average	-4.54 ± 1.86

AKT2 minus average	7.53 ± 4.37

PKC-α minus average	-5.20 ± 2.83

Values are the differences from the average of the four gene parameters in Table 1. Any set of three of the four parameters is an independent set. The fourth is a dependent parameter, since it is a linear combination (i.e. the negative sum) of the three parameters chosen to be independent. Percent standard errors were derived from the SVD variance-covariance matrix.

Values of predicted inhibition parameters for antisense S-DNAs targeted to different cell types, and/or to mRNAs encoded by different genes, can be compared by adding or subtracting the appropriate values at the bottom of Table 1 from the combinations of NNN parameters for a given target mRNA sequence. If all of the target mRNA sequences being compared are for the same cell type and gene, then the use of these cell- and gene-specific parameters is unnecessary, since in this case they are simply constant offsets and do not affect relative values being derived.

### NN parameters do not fit the data

Individual data sets in our SVD analysis were subjected to statistical analyses using *in vivo *NN parameters (although as mentioned above the NN model did not fit our data), and also using *in vitro *NN free energy values ΔG°(37°C) for DNA:RNA hybrids, in comparison with the use of the *in vivo *NNN parameters from Table 1. The percent inhibition by each sequence in the data set was calculated as for Figure 1 from the NNN parameters in Table 1, and analogous calculations were performed using the *in vivo *NN parameters. For the *in vitro *NN calculations, the total free energy was calculated by summing the ΔG°(37°C) values derived by Gray (Table 1C in [6]) using data from Sugimoto et al. [15]. For all of the calculations, the 20-mer target mRNAs were assumed to be closed circular sequences. The results for the four largest data sets from the current work are shown in the first four rows of data in Table 5. With the exception of the data for PKC-α inhibition in T24 cells, the calculations from both sets of NN parameters were more poorly correlated with the experimental data than were calculations using the NNN parameters. The *in vitro *NN parameters appeared to be better correlated with data obtained using T24 cells than with data obtained using A549 cells, but it should be noted that exactly the same S-DNA sequences were used in experiments to inhibit CRAF1 inhibition in both cell lines. This supports the notion that the cellular context can affect the efficacy of antisense S-DNA inhibition in a non-nearest-neighbor fashion. The generally poor correlation of the *in vitro *NN predictions with our data was not obviously due to the fact that the experimental data were obtained with S-DNAs, since the relative thermal stabilities of S-DNA:RNA hybrids are similar to those of unmodified DNA:RNA hybrids of the same sequences [16].

**Table 5:** Fits of data sets with NNN or NN parameters.

		Fit with in vivo NNN parameters		Fit with in vivo NN parameters		Fit with in vitro NN parameters	
**Experimental data**	**No. of Oligos**	**r_NNN_**	**P_sig_**	**r_NN_**	**P_sig _(NN)**	**r_NN_**	**P_sig_**

CRAF1 (A549 cells)	26	0.636	<0.001	0.146	0.474	0.242	0.234

CRAF1 (T24 cells)	26	0.748	<0.001	0.493	0.010	0.403	0.041

BCL2 (A549 cells)	29	0.635	<0.001	0.148	0.441	0.231	0.229

PKC-α (T24 cells)	24	0.903	<0.001	0.130	0.544	0.639	<0.001

Published data PKC-α (A549 cells) [17]	20	0.561	0.010	0.716	<0.001	0.368	0.110

Correlation coefficients, r, and the significance of correlation, P_sig_, between the indicated data sets and *in vivo *next-nearest-neighbor (NNN) parameters or *in vivo *or *in vitro *nearest-neighbor (NN) parameters. P_sig _is the significance of the correlation coefficient by the t-test and is the probability of being wrong in rejecting the null hypothesis. The smaller the value of P_sig_, the more significant the correlation.

It is not surprising that the calculated reductions in net protein accumulation using the NNN parameters fit the experimental data from which they were derived (Table 5). However, these parameters were not generally adequate to fit data from the literature in which antisense S-DNAs were used to inhibit the synthesis of a cellular protein or mRNA. Four sets of published inhibitory data in which 20 or more S-DNA sequences were used were compared with calculations from the NNN parameters and from both sets of NN parameters. The fits were poor, and in only one case was the correlation coefficient above 0.5 for calculations with any of the three parameter sets. In this case, the *in vivo *NNN and *in vivo *NN parameters were able to fit the inhibition data for PKC-α in A549 cells by Dean et al. [17]; results are shown in the last row of Table 5. Other sets of published data tested were: (1) 33 northern blot experiments for inhibition of expression of mRNAs for adhesion molecules (E-selectin and VCAM-1) in HUVEC cells [18], (2) 28 experiments for inhibition of COL1A1 collagen expression in mouse NIH 3T3 fibroblasts [19], and (3) 33 experiments for inhibition of angiotensin type-1 receptor levels in CHO cells [20].

## Discussion

One outcome of our study, in agreement with that of others [11], is that nearest-neighbor parameters, whether derived from *in vivo *data or from *in vitro *thermodynamic data, are not sufficient to fit sequence-dependent antisense data derived from *in vivo *cell culture experiments. Sequence effects that extend beyond those of the nearest-neighbors are likely to have numerous origins, adding to the complexity of predicting effective antisense targets mentioned in the Background. One source of these effects might involve sequence preferences of the binding sites of proteins that either inhibit or activate antisense inhibition, and these in turn could be influenced by the chemistry of the antisense DNA. For example, phosphorothioate-modified oligomers have been shown to bind with high affinity to single-strand DNA binding proteins and cellular proteins [21], while 2'-*O*-methyl modified RNAs have reduced non-specific protein binding and higher affinity for complementary RNAs [22-24]. It would be interesting to see how derived NNN and NN *in vivo *parameters depend on the use of different antisense DNA chemistries in which non-specific effects are reduced.

For this work, we considered it preferable to use experimental values that were all obtained by the same method in our own laboratory and yet provided a database large enough to demonstrate the NNN method of extracting sequence-dependent parameters. Therefore, a relatively small experimental database of 112 antisense experiments was used to derive the next-nearest-neighbor protein inhibition parameters summarized in Tables 1 and 2. The NNN parameters we derived were generally not valid predictors of the most effective targets reported in other published antisense experiments. This may reflect the plethora of effects of phosphorothioate-modified DNAs and/or the use of particular cellular systems and techniques. NNN parameters derived from experiments with second and third generation antisense oligomers [1] could be more widely applicable.

From our data the all-purine independent sequences had larger inhibitory parameters than did the all-pyrimidine independent sequences (Table 3), and this is consistent with the finding that some mRNA motifs such as GGGA, originally identified by Tu et al. [9], are purine-rich and are associated with effective antisense mRNA targets. In fact, there were 16 occurrences of the GGGA motif in the mRNA targets that ranked in the top 50% (most inhibited) of our experiments and only 4 in mRNA targets among the bottom 50% (least inhibited). However, there is no explicit relationship between the NNN independent sequence parameters derived in the present work and isolated tetrameric motifs, since the former include the contributions of overlapping sequences. As an example of the contrasting information in motifs and in a set of NNN parameters that include the contributions of overlapping sequences, consider the mRNA motif GGGA. A 12-mer sequence (GGGA)_3 _with three adjacent GGGA segments would have an inhibition parameter P of 23.0 ± 6.0% according to our SVD analysis and would rank in the middle of the values for the set of independent sequences in Table 2. However, the inhibition parameter can be very different for other 12-mer sequences. A higher P value of 46.8 ± 7.4% is calculated for (GGGAUC)_2_, and a value of only 4.4 ± 7.5%, an order of magnitude lower, is calculated for (GGGAGU)_2_. That is, a motif like GGGA may be part of a very favorable antisense target sequence, but just noting the presence of a GGGA motif, without considering the contributions of overlapping NNN within the full target sequence, overlooks additional information that determines how favorable that given target sequence might actually be.

It should be noted that sequences such as (GGGA)_3 _that are not listed among the linearly independent set in Table 2 are linearly dependent combinations of sequences such as (GGG)_4 _and (AGG)_4 _that are in Table 2, and consequently they have parameters that are linear combinations of those in the table. Thus, parameter P for (GGGA)_3 _may be calculated as P(GGGA)_3 _= (1/4) × P(GGG)_3 _+ (3/4) × P(AGG)_4 _= (1/4) × 24.8 + (3/4) × 22.4 = 23.0%. Parameters for more complex dependent sequences may be tedious to derive from those in Table 2, but they are easy to calculate by adding the values of the NNN parameters in Table 1 to give an identical result.

A final point is that, once derived, the parameters for NNN are straightforward to apply to calculate ranked inhibitory values for any other sequence. If the database includes measurements for different gene mRNA targets, cell lines, DNA chemistry, and other experimental variables such as exon/intron regions of the mRNA, these potentially can be included in the SVD analysis to provide offset parameters as illustrated in Table 4 for the different genes targeted in the present work. Aspects of new mRNA targets that are not part of the experimental database, such as accessible regions of the mRNA secondary structure and the uniqueness of the target sequence within the genome, would have to be considered as additional steps in the design of effective antisense DNAs.

## Conclusions

Retrospective analysis of actual sequence-dependent antisense inhibition data can provide useful information for the selection of mRNA targets and the subsequent design of effective antisense DNA sequences. We have shown that the sequence dependence of antisense inhibition extends beyond that of interacting, adjacent nearest-neighbors in the nucleotide sequence of the mRNA target (or antisense DNA oligomer). Extracting the maximal information requires taking into account the effects of overlapping sequences, such as overlapping NNN triplets that share common NN doublets. The simplest fashion in which this can be done is the extraction of NNN parameters by SVD, and the parameters can then be easily summed to evaluate the antisense potential of any sequence combination. The use of these parameters allows the percent reduction in net protein expression to be predicted for given mRNA target sequences, and the prediction includes all of the information for the particular distribution of NNN triplets in that sequence. This method allows that sequence to be uniquely ranked among all others with different NNN triplet combinations. This is unlike the ranking of sequences based on their contents of positive and negative inhibitory motifs. Standard errors are also provided by an SVD analysis, while errors are not readily available for combinations of motifs.

We have also shown that SVD analysis can include other information about the target sequence, antisense DNA chemistry, or experimental protocol that might affect the inhibitory potential of the mRNA target sequences in the database. There were larger reductions in the accumulation of protein (≈ 10%) using the A549 cell line than when the T24 cell line was used. Targets within the CRAF1 and AKT2 mRNAs were more successfully exploited than those within the BCL2 and PKC-α mRNAs, by differences of between 7 and 13% (Table 4). This latter difference did not seem to be dependent on the region of the mRNA being targeted, since about 50% of the target sequences were within the coding regions of the CRAF1, AKT2, and BCL2 mRNAs (12 of 26, 4 of 7, and 14 of 29, respectively) and the remainder were outside the coding regions. (All but one of the target sequences for PKC-α were in the coding region.) It is possible that these cell and target-specific parameters reflect differences in mRNA target secondary structures, lifetimes of the mRNA or protein, the presence of competing off-target sites, oligomer uptake, and/or other intracellular differences. Most relevant for the present work is that using sets of experiments that differ in their mRNA target sites, cell lines, or other aspects need not prevent the derivation of a consistent set of NNN triplet parameters. Moreover, the offset parameters themselves could lead to an appreciation of the magnitudes of effects that are secondary to the calculated sequence dependence of antisense DNA inhibition of protein expression. In the present work, the use of different cell lines and mRNA gene targets influenced the inhibitory efficacy of the antisense S-DNAs by about 10% within a range of from 11 to 66% inhibition of net protein accumulation.

## Methods

### Data set

Our analysis used a data set of 112 measurements of the reduction by antisense S-DNA oligomers of net intracellular protein synthesis (protein synthesized minus protein degraded). Data were obtained as described below for the antisense inhibition of CRAF1 protein accumulation in human A549 lung carcinoma cells and T24 bladder carcinoma cells (26 experiments each), AKT2 protein in A549 cells (7 experiments), BCL2 protein in A549 cells (29 experiments), and PKC-α protein in T24 cells (24 experiments). Percentage reductions in the net accumulations of these proteins after antisense DNA treatments are available in additional file 1.

### Antisense DNAs

20-mer antisense oligomer DNA sequences with phosphorothioate linkages (S-DNAs) were purchased from the Midland Certified Reagent Company (TX) and Oligos Etc. (OR). Sequences were selected to have complementarity to coding or to 3' or 5' non-coding regions of the mRNA (but not to introns), to have minimal base complementary in the human genome outside of the gene target of interest (by BLAST searches), to have no more than six adjacent self-complementary bases (since there is a negative correlation between intrastrand DNA nucleotide pairing and antisense efficacy [3]), and to have no more than three adjacent G's to minimize the possibility of G-quadruplex formation. The G+C content of the S-DNAs ranged from about 30 to 85%. Using a simple nearest-neighbor model, the predicted stabilities of DNA:RNA hybrids that would be formed with these sequences [14,15] ranged from a ΔG (37°) of -19 to -42 kcal/mol and were highly correlated with the percentage G+C content (correlation coefficient 0.94). Thus, the oligomers were chosen to have a wide range of hybridization stabilities. In previous work, we have shown that the melting temperatures of S-DNA:RNA hybrids rank closely with those of unmodified DNA:RNA hybrids of the same sequences [16].

### Antisense treatment of cell lines

Following procedures essentially the same as in previous publications [23,25], human A549 lung carcinoma and T24 bladder carcinoma cells were respectively cultured in McCoy's or RPMI media (Gibco BRL, MD) in the presence of 10% fetal bovine serum. The cells were transfected with 20-mer phosphorothioate DNA oligomers at 0.5 μM, pre-mixed with lipofectin (Life Technologies, CA) at a ratio of 1 lipofectin positive charge per 1.5 to 2 DNA phosphates. Following transfection for 4 hours, the transfecting solution was removed and the cells were incubated for a further 20-24 h in fresh medium. Cells were harvested and lysed in 1% (v/v) Triton X-100 or NP40 detergent, 1 mM dithiothreitol, 0.02% (w/v) sodium azide, 5-10% protease inhibitor cocktail (Sigma, MO), 0.15 M NaCl, and 0.05 M Tris-HCl, pH 8.0. Protein quantities in the lysates were quantitated using Bradford assays (Bio-Rad, CA) and 30 micrograms of protein from each lysate were electrophoresed in 7% or 12% polyacrylamide gels. In each case, the gel density was adjusted until controls showed complete removal of blotted proteins from the gel.

We were able to obtain significant reductions in PKC-α accumulation without the necessity of a phorbol pre-treatment as used by Dean et al. [17] to reduce the quantity of PKC-α protein present in cells prior to antisense oligomer treatment. Our results were consistent with the PKC-α protein having a half-life at the lower end of the reported range of 6.7-24 h [17]. Also, the inhibition of PKC-α protein accumulation was not altered by the presence of serum in some of our experiments (used to prevent apoptosis due to serum deprivation).

### Protein quantitation

The actual amounts of protein in the gel wells were quantitated by cutting the gel across the lanes and staining of the region of the gel containing actin with Coomassie Blue or Sypro stain. The remaining portion of the gel, containing the protein of interest, was blotted for 50 or 60 min onto Immobilon-P polyvinylidene fluoride membranes (Millipore, MA). Controls demonstrated no more than 1-5% leakage of protein from the first membrane onto a second membrane placed behind it. Immunoprobing of blotted membranes with antibodies to CRAF1 (BD Transduction Labs, CA), AKT2 (Santa Cruz, CA), BCL2 (Santa Cruz, CA), or PKC-α (Upstate Biotechnology/Millipore, MA) was followed by treatment with alkaline phosphatase-conjugated secondary antibodies (Jackson ImmunoResearch, PA), extensive washing, incubation with enhanced chemifluorescent substrate reagent (GE Healthcare, NJ), detection of fluorescence in a Molecular Dynamics STORM phosphorimager, and quantitation using ImageQuant software (version 5.0, GE Healthcare NJ). Further details are in previous publications [23,25].

### Next-nearest-neighbor data analysis

Following the procedures for the analysis of nearest-neighbor properties of hybrid duplexes [6,14], the set of 112 data points for the percent reduction of net accumulated protein by 20-mer antisense S-DNA oligomers was analyzed by singular value decomposition (SVD) to determine the relative importance of next-nearest-neighbor triplet combinations in the target mRNA sequence for hybridization with an antisense S-DNA oligomer. The next-nearest-neighbor triplets in a sequence, compared with nearest-neighbor base pairs, are illustrated in additional file 2. To simplify the consideration of NNN at the ends (i.e. to eliminate end triplets ENN and NNE', where N is any base, in the notation of Goldstein & Benight [26]), the assumption was made that each of the 20-mer mRNA target sequences was a closed circular sequence. This meant that the two end triplets were each considered to be composed of two NN, one correct and one approximated as if the two end nucleotides were adjacent. The resulting error amounted to 5-10%, depending on whether the inhibitory properties at the ends were dominated by the NN or NNN (i.e. 1/20 NN or 2/20 NNN are involved in closing a circular sequence). With this assumption, it was possible to derive parameters for 49 independent target sequences from our limited data set.

For the SVD analysis, each of the mRNA target sequences was separated into its 20 constituent NNN triplets, and the numbers of each of the possible 64 NNN for that target sequence were arrayed in one row of a matrix. The 86 target sequences for which the reductions in accumulated protein were determined (26 of which were used in both A549 and T24 cell lines) thus gave 112 rows in a 112 row × 64 column matrix. An example 3 × 64 matrix for three CRAF1 sequences is illustrated in additional file 3. In addition, five extra columns were added to the matrix, one of which was used to designate the cell type (with the number 1 in the column if the experiment with that target sequence used A549 cells), and four of which were used to designate the targeted gene (with the number 1 in the column of the gene specific to that mRNA). The final result was a 112 row × 69 column matrix. The 64 NNN were all generally well represented in the data base, averaging 35 ± 16, with only four NNN being present less than 15 times (CUA, UAG, UUA, and UAA were represented 14, 11, 10, and 8 times, respectively; see Table 1, last column). Each row of values was divided by the error for that experiment to give the matrix of values, **N**_h, k _(h = 1...112, k = 1...69). Experimental values of percent reductions in accumulated protein for the 112 antisense experiments (divided by their respective errors) were arrayed in a column vector I_h_. The matrix equation **N**_h, k _× P_k _= I_h _was then solved for the vector array P_k _of 69 inhibition parameters for the 64 NNN, 4 different gene targets, and cell line, using SVD as described by Press et al. [13] and in our previous work [6].

Following the reasoning described by Gray [14], there are only 49 linearly independent combinations of the 64 NNN because there are 15 constraints on arranging the NNN when they are in a closed circular sequence. There was also a relationship among the columns of the matrix that specified which genes are targeted, in that their sum was constrained to 1. Therefore, the **N**_h, k _matrix was a singular matrix with 16 singular values of essentially zero (10^-5 ^less than the smallest significant singular value). Only five of the 69 parameters describing the reduction in accumulated protein were directly meaningful, those for the triplet NNNs of AAA, UUU, GGG, and CCC, and for the cell type. However, the significance of the remaining triplet NNNs could be meaningfully described in terms of the values for independent sequence combinations, and gene combinations, just as if the parameters for the independent combinations were directly derived from a nonsingular matrix [13,14].

## Competing interests

The authors declare that they have no competing interests other than that DMG is inventor of US Patent No. 6,957,148 relating to the content of the manuscript.

## Authors' contributions

DMG conceived of the study, performed analyses, and drafted the manuscript.

CWG developed a standardized method for quantitating the net accumulation of synthesized proteins.

Antisense inhibition data were obtained by CWG (for PKC-α in T24 cells), BHY (for AKT2 and BCL2 in A549 cells), and TFL (for C-RAF1 in A549 and T24 cells).

All authors have read and approved the final manuscript.

## Acknowledgements

Support was provided by grants AT-503 from the Robert A. Welch Foundation, 00741-0021-1999 from the Texas Advanced Technology Program, and from eXegenics, Inc., of Dallas, TX. Preliminary results were reported in US Patent No. 6,957,148 and in the PhD dissertation of BHY (The University of Texas at Dallas, 2004).

## Supplementary Material

Additional file 1 mRNA sequences targeted by antisense S-DNAs and percent reduction in protein accumulation. Click here for file

Additional file 2 Example of nearest-neighbor and next-nearest-neighbor base pairs. Click here for file

Additional file 3 Example matrix of next-nearest-neighbor triplets. Click here for file
