# Supplementary material for: Antisense DNA parameters derived from next-nearest-neighbor analysis of experimental data
Source: BMC Bioinformatics. 2010 May 14;11:252. doi: 10.1186/1471-2105-11-252 (PMC2877693; doi:10.1186/1471-2105-11-252)
Supplement: Additional file 1 — mRNA sequences targeted by antisense S-DNAs and percent reduction in protein accumulation. [file 1471-2105-11-252-S1.PDF]

Additional file 1. mRNA sequences targeted by antisense S-DNAs and percent reduction in protein accumulation.

Gene	Sequence starting position in mRNA of 20-mer target	% Reduction of protein level in T24 cells	Error (%)**	% Reduction of protein level in A549 cells	Error (%)**
CRAF1	1	37	10	26	** *
	41	47	5	48	** *
	61	53	14	30	** *
	85	39	16	41	10
	121	53	1	50	** *
	130	33	12	43	10
	181	37	3	45	** *
	301	34	** *	32	** *
	361	39	** *	38	** *
	707	35	13	22	11
	761	29	7	45	10
	821	43	7	51	10
	1041	36	11	48	1
	1063	48	19	49	11
	1181	57	0	54	10
	1474	48	12	52	7
	1777	40	13	52	11
	1867	43	11	56	6
	2098	45	12	46	12
	2341	66	25	60	0
	2349	36	11	37	11
	2484	59	20	52	5
	2581	57	15	63	3
	2601	24	2	42	13
	2661	42	9	66	1
	2681	42	2	49	4
AKT2	57			41	14
	86			54	5
	94			46	6
	490			58	7
	973			55	9
	1188			28	13
	1227			41	17
BCL2	23			41	14
	36			54	5
	61			46	6
	109			58	7
	120			55	9

	201			28	13
	277			41	17
	316			51	6
	361			30	14
	409			46	8
	445			53	8
	453			45	3
	501			35	9
	541			34	12
	601			28	11
	1041			50	5
	1421			53	8
	1481			56	5
	1641			49	10
	1761			52	5
	1821			28	10
	1941			50	10
	2081			43	10
	3101			37	6
	3921			41	6
	3941			43	4
	3961			42	6
	4881			44	7
	5321			45	4
PKC-α	121	42	9		
	281	52	10		
	288	47	5		
	301	22	9		
	321	24	2		
	341	29	4		
	421	14	18		
	441	17	10		
	481	11	3		
	501	54	10		
	541	15	1		
	621	23	11		
	681	25	6		
	741	28	9		
	761	42	3		
	801	22	3		
	841	41	5		
	881	27	3		
	899	53	6		
	1001	23	11		
	1021	25	1		

	1081	33	6		
	1181	18	6		
	2044	42	1		

Four genes in either T24 or A549 cell lines were targeted by a total of 86 different 20-mer phosphorothioate DNAs. Of these, 26 S-DNAs were used to treat both cell lines for inhibition of CRAF1 protein accumulation, giving a total of 112 data points.*

* The % reductions of protein levels for five additional S-DNAs targeted to CRAF1 mRNA in one or both cell lines in a preliminary NNN analysis had squared deviations of at least 20× the average of inhibition data for other sequences, and these five sequences were omitted from the final set.

** Errors are ranges from duplicate Western blots or standard deviations from three or more measurements

*** Where no error is shown, the data were from single measurements and for the purpose of SVD analysis a maximum error of 15% was assumed.
