# Supplementary material for: Antisense DNA parameters derived from next-nearest-neighbor analysis of experimental data
Source: BMC Bioinformatics. 2010 May 14;11:252. doi: 10.1186/1471-2105-11-252 (PMC2877693; doi:10.1186/1471-2105-11-252)
Supplement: Additional file 2 — Example of nearest-neighbor and next-nearest-neighbor base pairs. [file 1471-2105-11-252-S2.PDF]

Additional file 2. Example of nearest-neighbor and next-nearest-neighbor base pairs.

(A)

```
20-mer DNA:     5'X-X-X-X-X-X-X-G-T-A-C-X-X-X-X-X-X-X-X-X 3'
mRNA:      3'....-x-x-x-x-x-x-x-C-A-T-G-x-x-x-x-x-x-x-x-x-....5'
```

(B)

```
20-mer DNA:     5'X-X-X-X-X-X-X-G-T-A-C-X-X-X-X-X-X-X-X-X 3'
mRNA:      3'....-x-x-x-x-x-x-x-C-A-T-G-x-x-x-x-x-x-x-x-x-....5'
```

(A) Three nearest-neighbor base pairs are boxed. Each nearest-neighbor is independent in that the stacking interactions between adjacent base pairs do not overlap. (B) Two next-nearest-neighbor base pairs are boxed. Each of these have an adjacent set of stacked base pairs in common (the 5'T-A3'/5'T-A3' stacked pair).
