# Supplementary material for: Antisense DNA parameters derived from next-nearest-neighbor analysis of experimental data
Source: BMC Bioinformatics. 2010 May 14;11:252. doi: 10.1186/1471-2105-11-252 (PMC2877693; doi:10.1186/1471-2105-11-252)
Supplement: Additional file 3 — Example matrix of next-nearest-neighbor triplets. [file 1471-2105-11-252-S3.PDF]

Additional file 3. Example matrix of next-nearest-neighbor triplets.

Matrix NNN triplets 1-16:

	1	2	3	4	5	6	7	8	9	10	11	12	13	14	15	16
mRNA Seq	AAA	AAU	AAC	AAG	AUA	AUU	AUC	AUG	ACA	ACU	ACC	ACG	AGA	AGU	AGC	AGG
#85	0	0	0	0	0	0	0	0	0	0	0	0	0	0	0	1
#130	0	0	0	0	1	0	0	1	2	0	0	0	0	0	2	1
#707	1	1	0	0	0	2	0	0	0	0	0	0	0	0	0	0

Matrix NNN triplets 17-32:

17	18	19	20	21	22	23	24	25	26	27	28	29	30	31	32
UAA	UAU	UAC	UAG	UUA	UUU	UUC	UUG	UCA	UCU	UCC	UCG	UGA	UGU	UGC	UGG
0	0	0	0	0	0	0	0	1	0	1	0	0	0	0	1
0	0	1	0	0	0	0	0	0	0	0	0	0	0	0	1
0	1	0	0	1	1	2	1	0	1	2	0	0	1	0	0

Matrix NNN triplets 33-48:

33	34	35	36	37	38	39	40	41	42	43	44	45	46	47	48
CAA	CAU	CAC	CAG	CUA	CUU	CUC	CUG	CCA	CCU	CCC	CCG	CGA	CGU	CGC	CGG
0	0	0	1	0	0	2	1	0	2	1	0	0	0	0	1
0	2	1	1	0	0	0	0	0	0	0	0	0	0	0	0
1	0	0	0	0	1	1	0	1	1	0	0	0	0	0	0

Matrix NNN triplets 49-64:

49	50	51	52	53	54	55	56	57	58	59	60	61	62	63	64
GAA	GAU	GAC	GAG	GUA	GUU	GUC	GUG	GCA	GCU	GCC	GCG	GGA	GGU	GGC	GGG
0	0	0	0	0	0	0	0	0	1	1	1	0	0	3	2
0	0	0	2	0	0	0	0	2	0	0	0	2	0	0	1
0	0	0	0	0	1	0	0	0	0	0	0	0	0	0	0

Values in this example matrix of dimensions 3×64 show the occurrences of the 64 possible next-nearest-neighbor triplets for three 20-mer mRNA sequences in CRAF1 mRNA. The sequences are considered to be closed circles, so each sequence has 20 NNN triplets. Sequences

are #85: 5'-GGCGGCCUGGCUCCCUCAGG-3', #130: 5'-AUGGAGCACAUACAGGGAGC-3', and #707: 5'-UCUUAUUGUUUCCAAAUUCC-3'.
